# On the Difference of Scoring in Speech in Babble Tests

**DOI:** 10.3390/healthcare10030458

**Published:** 2022-02-28

**Authors:** Afroditi Sereti, Christos Sidiras, Nikos Eleftheriadis, Ioannis Nimatoudis, Gail D. Chermak, Vasiliki Maria Iliadou

**Affiliations:** 1 Clinical Psychoacoustics Laboratory, 3rd Psychiatric Department, Neurosciences Sector, Medical School, Aristotle University of Thessaloniki, 54124 Thessaloniki, Greece; chsi@mmmi.sdu.dk (C.S.); jnimatoudis@auth.gr (I.N.); viliad@auth.gr (V.M.I.); 2 Faculty of Engineering, The Maersk Mc-Kinney Møller Institute, University of Southern Denmark, 5230 Odense, Denmark; 3 ENT, Private Practice, 54623 Thessaloniki, Greece; nikeleft@auth.gr; 4 Department of Speech and Hearing Sciences, Elson S. Floyd College of Medicine, Washington State University Health Sciences Spokane, Spokane, WA 99202, USA; chermak@wsu.edu

**Keywords:** auditory processing disorder, speech in noise tests, Greek Speech-in-Babble test, hearing, cognition, word-based scoring, syllable-based scoring

## Abstract

Hearing is a complex ability that extends beyond the peripheral auditory system. A speech in noise/competition test is a valuable measure to include in the test battery when attempting to assess an individual’s “hearing”. The present study compared syllable vs. word scoring of the Greek Speech-in-Babble (SinB) test with 22 native Greek speaking children (6–12-year-olds) diagnosed with auditory processing disorder (APD) and 33 native Greek speaking typically developing children (6–12-year-olds). A three-factor analysis of variance revealed greater discriminative ability for syllable scoring than word scoring, with significant interactions between group and scoring. Two-way analysis of variance revealed SinB word-based measures (SNR50%) were larger (poorer performance) than syllable-based measures for both groups of children. Cohen’s d values were larger for syllable-based mean scores compared to word-based mean scores between groups for both ears. These findings indicate that the type of scoring affects the SinB’s resolution capacity and that syllable scoring might better differentiate typically developing children and children with APD.

## 1. Introduction

Hearing in everyday life requires skills that go beyond auditory sensitivity as measured by the pure tone audiogram [1]. The term auditory processing disorder (APD) refers to a specific auditory deficit along the central auditory nervous system, including bottom-up and top-down neural connectivity [2]. Central auditory processing disorder is currently classified in the International Statistical Classification of Diseases and Related Health Problems, 10th revision (ICD-10) as H93.25. Children with APD present with a wide range of auditory symptoms, most commonly difficulties understanding speech in background noise [3,4]. APD is associated with a range of functional deficits, including educational and psychosocial challenges [5,6]. The disorder is evaluated by psychoacoustic/behavioral tests as well as electrophysiological procedures [7]. APD is included in the World Health Organization’s (WHO) recent report on hearing [8]. The WHO highlights the presence of APD across the lifespan. While current audiological test batteries offer a relatively high degree of sensitivity and specificity in correctly diagnosing APD, approaches to improve efficiency, particularly in non-English languages, are needed.

Given the high prevalence of speech in noise processing deficits in APD [3,4], as well as the speech recognition in noise difficulties experienced by many individuals with sensorineural hearing loss, testing this specific auditory skill should be an integral part of audiological test batteries. A recent European consensus of 17 countries [2] (that is currently expanding to include additional countries) recognizes that clinical audiological practice rarely goes beyond hearing sensitivity testing in Europe. The complexity of auditory processing evaluation, requiring informed selection of tests to be administered along with careful interpretation of results, often leads some to avoid going beyond the audiogram and objective (i.e., electroacoustic and electrophysiological) audiology measures [9]. It should be kept in mind that differential diagnosis is crucial, since auditory processing deficits may coexist with other disorders (e.g., language, cognitive, and/or psychiatric disorders) leading to bidirectional influences and confounding and exacerbating effects [10]. Auditory processing evaluation includes both verbal and non-verbal testing to provide more comprehensive information on how the auditory system transmits auditory stimuli to the brain [11].

As speech in noise recognition tests have been developed in the past decades for different languages, two different types of speech material have been most commonly used (i.e., whole sentences [12,13] and single words [14,15,16], although syllables have been used in research on dichotic listening) [17]. Sentences better simulate everyday hearing conditions [18,19]. However, recognizing sentences imposes greater language and cognitive processing, possibly introducing confounding factors, such as syntax processing and working memory [20,21,22,23,24].

Theories and models of speech processing offer a perspective regarding the use of different stimuli in speech recognition testing. Speech consists of a continuous signal that can be parsed into separate chunks at different linguistic levels (i.e., phonemes, syllables, words etc.) [25,26,27]. Infants are able to segment speech by eight months of age [28], presumably facilitated by the prosodic information of the incoming auditory signal [29]. Additionally, speakers tend to segment the speech signal based on stress patterns (e.g., the existence of strong vs weak syllables). In particular, speakers of English are more likely to treat strong syllables as the initial syllables of lexical words (and less likely to do so when weak syllables appear) [29,30]. Prosody, a suprasegmental feature that is important for communication and learning, is linked to speech rhythm [9]. Rhythm facilitates speech in noise recognition in typically developing children [31] through neural synchronization of auditory information processing. This facilitation may not be present in children diagnosed with APD [32]. Even though young children are able to discriminate and manipulate syllables as separate units (i.e., phonological processing), the same skills are not present at the phonemic level (i.e., phonemic awareness) until children develop literacy skills in the early primary school years [33].

Syllable and phoneme segmentation are executed by the central auditory nervous system, specifically at the auditory cortex [34,35]. This mechanism consists of at least two separate levels of neural oscillatory activity, nested within each other, which correspond to the syllables and phonemes [34,36]. The interplay between these low levels of sensory processing of speech suggests that taking into account the recognition of small chunks separately in assessing speech in noise recognition tests, instead of merging them into larger units, may offer a more sensitive and accurate assessment of the individual’s abilities compared to larger linguistic units, like sentences. This can be implemented without imposing the greater cognitive and linguistic demands of sentences by using words as stimuli and/or by scoring syllables instead of the whole word (at least in languages with multisyllabic content words as is the case in the Greek language where there are few monosyllabic content words) [37]. It should be kept in mind that the aforementioned constraint, namely the scarcity of monosyllabic content words in the Greek language, together with the requirement of choosing phonetically balanced words for word lists, led to the use of bisyllabic words in the Greek Speech-in-Babble test (the SinB) described below [37].

The Speech-in-Babble (SinB) test is a Greek speech in noise recognition test that uses bisyllabic words as speech material [15,16]. Performance on the SinB is calculated as the signal-to-babble ratio at which 50 percent of the words are correctly repeated. The tool was initially tested in normally hearing adult Greek native speakers (N = 37) with a mean age 24.4 (years: months) and a standard deviation of 6.2 years. Audiometric testing for all participants for frequencies 0.5, 1, 2, and 4 kHz was completed before administering the SinB. Despite its initial administration with an adult population, the tool has been used successfully to test children for the diagnosis of auditory processing disorder [16] and has also been found to be highly reliable for screening purposes in differentiating between APD and typically developing participants in the laboratory and in practice [38]. In the present study the SinB has been used as a diagnostic tool.

The primary purpose of speech in noise recognition tests is to assist in diagnosis and evaluation. This is achieved by establishing cut-off values according to performance of an age-matched population with normal peripheral auditory function, beyond which performance is considered below norms. Scoring can consist of a simple percent correct or employ a more statistical approach (e.g., Spearman–Karber formula). This formula determines the signal-to-noise ratio (SNR) at which 50% of the stimulus material is correctly recognized or SNR50% (Spearman–Karber formula (50% correct performance = i + 1/2(d)−(d) correct score/w, where i is the initial SNR, d is the step size of presentation level, and w is the number of items per decrement (per step)). Note that the unit that comprises an item is not specified, and thus the formula can be applied to a whole sentence, a phrase, a word, or a single syllable.

The authors are aware of no research that has specifically examined the effects of differing stimulus length or complexity of scoring on the SinB. Although scoring of speech in babble tests in English has been evaluated [39], scoring approaches in languages other than English are scant. The purpose of the present study was to compare the relative efficiency of syllable-based scoring and word-based scoring of the Greek SinB in differentiating children with APD vs typically developing children. This was achieved by administering the SinB, calculating both syllable and word scores from this single administration, and submitting both types of scores for each participant for statistical analysis.

## 2. Materials and Methods

### 2.1. Study Design

SinB was administered as part of a diagnostic test battery for children seen for APD evaluation in the University Hospital of Thessaloniki Psychoacoustics Clinic. Both standard word-based and syllable-based scores were calculated from the same test administration. Several statistical approaches were employed to examine whether the novel syllable-based scoring approach improves the efficiency of the SinB in differentiating the children with APD from the typically developing children (see section ‘Statistical Analysis’).

### 2.2. Participants

Twenty-two children diagnosed with APD (mean age = 8.3, SD = 1.7, range = 6–12 years) and thirty-three typically developing age-matched children (mean age = 9.2, SD = 1.7, range = 6–12 years) participated in this study. The inclusion criteria for the control group were: (a) age-appropriate writing skills (according to teachers’ reports), (b) Greek as first language, and (c) no known or suspected developmental disorder (as reported by both teachers and caregivers). Participants of both groups were native Greek speakers and matched for age range. Analysis of variance (ANOVA) showed no statistically significant difference in age range between groups (*F*(1, 54) = 3.38, *p* > 0.05). All participants presented normal hearing sensitivity bilaterally as revealed by pure-tone averages (PTA) of 15 dB HL or better at all octave frequencies between 0.25 and 8 kHz. Children with APD were referred for listening and academic problems by speech pathologists and/or educators and were diagnosed with APD in the Psychoacoustic Clinic in the AHEPA University Hospital of Thessaloniki. Diagnosis was made using a clinical psychoacoustic test battery customized to each participant based on case history, including the Speech in Babble test (SinB) [11,16] using the standard word scoring, dichotic digits (DD) [40], forward (FDR) and backward digit recall (BDR) [41], random gap detection test RGDT [42], gaps in noise (GIN) [43,44], pitch pattern sequencing (PPS) [45], and duration pattern sequencing (DPS) [45]. Children with performance deficits of 2 SDs or greater from the mean of age-matched typically developing children on at least two tests (including at least one nonverbal test) in at least one ear were diagnosed with APD [2,46]. The average time for the diagnostic procedure was 45–60 min per child.

Parents and caregivers of both groups gave their written consent for participation in the study, in accordance with the World Medical Association’s Declaration of Helsinki to be evaluated for hearing sensitivity and auditory processing.

### 2.3. Testing

SinB [15,16] is a monaural speech in noise test of bisyllabic Greek words in which participants are instructed to repeat the word heard after each trial. Fifty bisyllabic words (ten at each SNR level) were presented in a background of multi-talker babble at a constant input level of 60 dB HL and a fixed order of five different signal-to-noise ratios (SNR; +7 dB, +5 dB, +3 dB, +1 dB, and −1 dB) for each ear. Each word was preceded by a short verbal attentional cue (“pite tin lexi,” i.e., “say the word”). SNRs at which 50% of the items were correctly identified were calculated using the Spearman–Karber formula ((50% correct performance = i + 1/2(d)−(d) correct score/w, where i is the initial SNR, d is the step size of presentation level, and w is the number of items per decrement (per step)). Higher scores (measured in dB SNR) reflect poorer performance; smaller scores (measured in dB SNR) reflect better performance.

For each participant, two scores were calculated for each ear based on one administration of the SinB. Word-based scores SinB_RE_w & SinB_LE_w (for right and left ears respectively) and syllable-based scores SinB_RE_s & SinB_LE_s (for right and left ears respectively). For word-based scoring, the number of items in the Spearman–Karber formula reflects correctly identified bisyllabic words. If the participant repeated only one of the two syllables presented, that word is scored as incorrect. For syllable-based scoring, the number of items in the Spearman–Karber formula is based on the number of correctly identified individual syllables. Therefore, if one syllable of the bisyllabic word is correctly recognized, the particular syllable is scored as correct and the non-recognized syllable as incorrect.

### 2.4. Statistical Analysis

Data were normally distributed based on the criteria of skewness and kurtosis, with z values ranging between −1.96 and +1.96 [47,48]. Hence, parametric statistics were used to analyze the data. SinB test’s efficiency (i.e., ability to differentiate the two groups) was assessed via ANOVA F values). Cohen’s d’ was used to examine effect size for the two scoring methods.

## 3. Results

**Figure 1:**
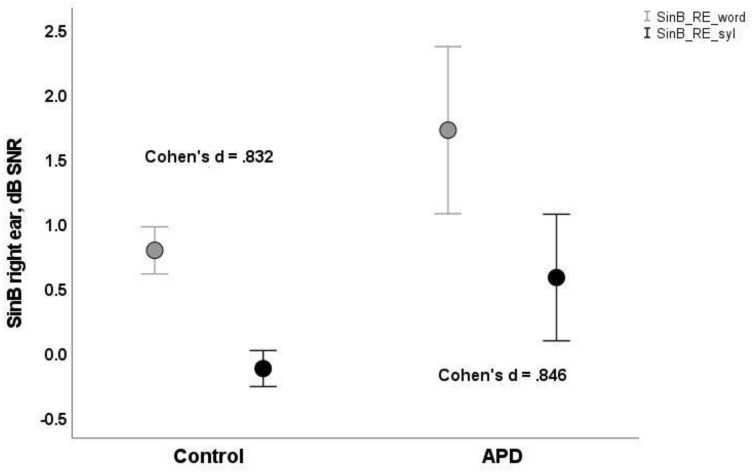
(SinB RE) Means and 95% error bars for SinB word (bright circles rectangles) and syllable-based scores (dark circles) for control and APD (Auditory Processing Disorder) children, right ear. Cohen’s d values refer to the effect size between typically developing and APD (Auditory Processing Disorder) groups for word and syllable-based scores.

**Figure 2:**
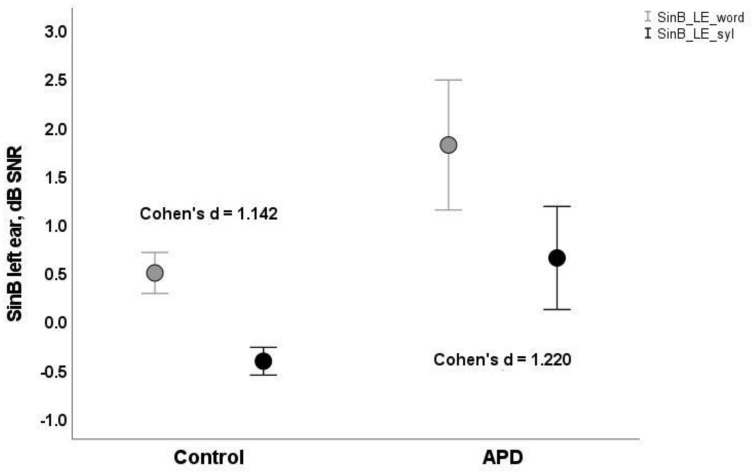
(SinB LE) Means and 95% error bars for SinB word (bright circles rectangles) and syllable-based scores (dark circles) for control and APD (Auditory Processing Disorder) children, left ear. Cohen’s d values refer to the effect size between typically developing and APD (Auditory Processing Disorder) groups for word and syllable-based scores.

Both groups of children performed more poorly (i.e., higher SNR50%) for word scoring than syllable scoring. APD children’s mean scores were higher (i.e., poorer) than typically developing children’s, both for word scoring (mean = 1.33, SD = 0.28 vs. mean = 0.93, SD = 0.11; mean = 1.43, SD = 0.29 vs mean = 0.55, SD = 0.09, for right and left ears respectively) and for syllable scoring (mean = 0.28, SD = 0.21 vs mean = -0.03, SD = 0.08; mean = 0.37, SD = 0.23 vs mean = −0.38, SD = 0.07; see Table 1 and Figure 1 and Figure 2).

A three-factor (2 × 2 × 2) ANOVA was used to test for statistical significance (Factors: group (APD vs typically developing), scoring type (word vs. syllable), ear (right vs left)). A statistical difference between group scores was identified (*F*(1, 54) = 18.9, *p* < 0.001), and syllable-based mean SinB scores were significantly lower (i.e., better) compared to word-based means (*F*(1, 54) = 657.1, *p* < 0.001). A significant interaction was revealed between group and type of scoring (*F*(1, 54) = 8.045, *p* = 0.006), indicating larger differences between groups for syllable-based compared to word-based scores. In addition, a significant group by ear interaction was seen (*F*(1, 54) = 5.1, *p* = 0.028). To further assess the group by ear interaction, separate 2 × 2 ANOVAs were run for each ear (Factors: group (APD vs typically developing) and scoring type (word vs. syllable)). Results revealed larger F values (i.e., larger differences between groups) for the left (*F*(1, 54) = 22.6, *p* < 0.001) versus the right ear (*F*(1, 54) = 12.2, *p* < 0.001).

Cohen’s d was used to test whether the effect size of the difference found between groups was significantly larger for the syllable scores versus the word scores. Cohen’s d values [49] were calculated for all four SinB scores to examine differences between groups using the formula:

$$Cohen^{\prime }s\;d=\frac{m_{2}-m_{1}}{\sqrt{\left(SD_{1}^{2}+SD_{2}^{2}\right)/2}}$$

where *m_1_* and *m_2_* are the means and *SD_1_* and *SD_2_* are the standard deviations of the two distributions, respectively. Larger values of Cohen’s d signify greater differences between distributions. Cohen’s d values were somewhat larger for syllable-based scores (s) relative to word-based scores (w) for both ears (RE_s = 0.846 vs RE_w = 0.832 vs.; LE_s = 1.220 vs. LE_w = 1.142 respectively) (See Table 1).

## 4. Discussion

This study was undertaken to examine the potential enhanced efficiency using syllable scoring of the SinB in differentiating performance between children with APD and typically developing children. Analysis revealed that both groups performed more poorly (larger SNR50%) for word-based vs. syllable-based scoring. However, syllable-based scoring yielded larger mean differences between groups.

Results suggest that there is an advantage in using syllable vs word-based scoring with children in clinical practice with the SinB, as the former resulted in larger effect sizes and modestly increased its efficacy in differentiating between typically developing participants and those with APD. This scoring technique can be thought of as a means of improving the test’s ‘resolution’ capacity, as it takes into account smaller units of speech information. Although English speech (word) in noise/babble recognition tests use monosyllabic words and scoring is not differentiated as each word consists of one syllable, other languages, like Greek, which have fewer monosyllabic words, may benefit from scoring based on segments smaller than words. The more detailed (for the audiologist) syllabic scoring approach may increase the discriminative value of the test as well as better reflect the individual’s real world, everyday speech recognition in noise function. Indeed, redundancy within the signal (including the potential internal redundancy of bisyllabic words) benefits real world speech recognition in noise/competition. One might argue that syllable scoring may better reflect that benefit than whole word scoring.

To better understand why the test’s resolution capacity increases when smaller phonological units are scored (syllables) instead of larger ones (words), one needs to consider that signal-to-noise or babble ratio varies throughout the SinB. This results in instances in which the competition (i.e., babble) is more intense than the signal (i.e., a negative SNR). Since some syllables will be more audible in competition than others due to fine spectral characteristics (e.g., ‘stiθos’ (‘breast) for ‘stixos’ (verse), since /θ/ and /x/ differ only in one phonetic feature (place of articulation)), syllable scoring reveals these fine differences that might be obscured in word scoring. Simply put, syllable-based scoring is more granular and precise, and this seems to increase its resolution capacity.

Syllable-based scoring also allows the clinician to examine the individual’s ability to recognize nonmeaningful speech (i.e., syllables that carry no lexical value) alongside syllables that are lexical, thereby adding another advantage to this scoring approach, particularly when testing those with language processing issues. The processing of non-speech stimuli is not constrained by linguistic features, since the processing of these stimuli depends on simple acoustic characteristics (i.e., frequency, intensity, and duration). The scoring of meaningful speech stimuli, on the other hand, is more demanding, engaging language and greater cognitive resources. Syllable scoring seems to be lying between behavioral speech recognition tasks measuring the processing of non-speech stimuli and behavioral tasks measuring the processing of more complex speech stimuli (e.g., phrases or sentences) offering a more detailed view of the complicated process that occurs while processing speech stimuli. Since single syllables (particularly in Greek) do not convey lexical information, syllable scoring may reflect Greek listeners’ processing of non-speech stimuli relative to word scoring that reflects the more complex processing of linguistic units.

Lower-level (syllabic) scoring provides greater granularity and likely explains why syllable scoring rendered the SinB more efficient in discriminating children with APD from typically developing children. One might anticipate that scoring even smaller speech segments (i.e., continuant phonemes) would further increase a test’s resolution capacity beyond syllable scoring. Both theoretically and practically, it would be of great interest and importance to have access to such detailed information, especially if we consider that most speech in competition tests score performance at the word level, losing significant details not only with regard to how many phonemes are recognized, but also (and even more importantly) with regard to which phonemes are recognized and what features those phonemes reflect (e.g., voicing, nasality, sibilance). Most speech recognition tests (with the exception of sentence tests in which the score is based on key word recognition) produce a unidimensional score. The use of a single number to describe the complicated process of speech recognition, particularly in competition, seems at least odd and overlooks underlying data that might improve diagnosis and treatment. Asking participants to identify phonemes might be more demanding in terms of temporal resolution, but it would provide a more limited set from which the correct answer exists (i.e., the number of phonemes in a language’s repertoire is considerably smaller than the number of syllables or words in a language). Thus, phoneme-based scoring could be more efficient. Moreover, multidimensional scoring might differentiate between a deficit in global phoneme identification versus deficits in a group of phonemes (or in a certain phoneme) that could be explained by configuration of hearing loss or site and/or degree of APD. For instance, it might be tempting to suggest that a temporal resolution impairment would affect the recognition of transient plosive consonants (e.g., /p/, /t/, /k/), but not non-transient consonants (e.g., /s/, /z/) or vowels. Similarly, speech requires a degree of processing in different parts of the central auditory nervous system that do not participate in processing non-linguistic input [50,51,52,53]

One could also consider scoring suprasegmental information, such as stress, which varies as a function of position of a syllable within a non-monosyllabic word and contributes to speech recognition. Sidiras et al. [32] took into account syllable position for a rhythm speech in noise test that combined bisyllabic words, babble, and rhythmic and non-rhythmic beat-sequences (word recognition—rhythm component, WRRC). Work preceding the WRRC test [31] suggested that rhythm has a non-uniform effect on speech in noise recognition across syllables and the magnitude of the effect is a result of the interaction between syllable position and presence versus absence of lexical stress. These findings highlight the need for a more elaborate scoring method (i.e., syllable scoring).

The present results support the use of syllable-based scoring of the SinB with children to improve its resolution and potential discriminative capability. Future research should examine the potential benefits of phonemic and possibly suprasegmental scoring (i.e., stress) with children and atypical populations. While such scoring is more demanding for the clinician and might be prone to greater error when patient responses are repeated (i.e., rather than written or pointing to pictorial representations), the use of more sophisticated scoring sheets and approaches to scoring (e.g., calculating one measure for all stop consonants, rather than one for each phoneme) might minimize these potential challenges. Moreover, phoneme scoring might decrease test time due to the increased number of scored tokens per word. The use of a phoneme scoring rubric developed by Billings et al. [39] provides one approach to implementing phoneme scoring in clinical practice with relative ease.

Increasing the efficiency of diagnostic tests is an ongoing focus of clinical research across disorders, including APD [9,54,55]. Relying on syllable scoring may reduce top-down influences of language and cognition and improve the sensitivity and specificity of speech recognition in competition tests. Indeed, assessment of speech recognition in competition is an integral component of APD diagnostic evaluations as it provides insight regarding the individual’s difficulty processing low-redundancy speech, known to be a hallmark of APD [56,57] and assesses an important functional skill of listening in noise or other background competition [4]. Syllabic scoring, which reduces the extrinsic redundancy of the stimulus, might increase the efficiency of speech recognition in competition measures, notwithstanding the poor sensitivity observed for the SinB across all conditions in the present study. In fact, Weihing et al. [58] reported that a commonly administered low-redundancy speech test (low-pass filtered speech) was one of the tests most commonly failed by children with APD. Improving existing speech in noise tests to expand the way we measure speech recognition [25] can be a promising pathway for gaining new diagnostic insights in populations presenting with a range of hearing issues including APD.

## 5. Conclusions

Speech recognition in noise/babble tests (i.e., monaural low-redundancy tests) are among the most frequently used tools in the test battery in clinical practice for the diagnosis of children with auditory processing deficits and, by extension, for differentiating between typically developing children and children with APD. Findings of this study indicate that the type of scoring affects the SinB’s resolution capacity. Syllable scoring compared to word scoring might better differentiate children with APD from their typically developing peers.

## Figures and Tables

**Table 1:** Mean scores and standard deviations of SinB (Speech-in-Babble test) measures (word and syllable based, right and left ear) for control and APD (Auditory Processing Disorder) group, and ANOVA and Cohen’s d.

		SinB_RE_w	SinB_RE_s	SinB_LE_w	SinB_LE_s
**Mean, SD**	**Control**	0.93 (0.11)	−0.03 (0.08)	0.55 (0.09)	−0.38 (0.07)
	**APD**	1.33 (0.28)	0.28 (0.21)	1.43 (0.29)	0.37 (0.23)
**ANOVA F, p**		11.19 * (0.002)	11.56 * (0.001)	20.84 * (0.001)	24.15 * (0.001)
**Cohen’s d**		0.832	0.846	1.142	1.220
**Repeated ANOVA F, *p***		6.81 * (0.012)		7.84 * (0.007)	
**Repeated ANOVA F, *p***		8.045 * (0.006)			

* means *p* is less than 0.05.

## Data Availability

The data presented in this study are available on request from the corresponding author.

## Abbreviations

APD	auditory processing disorder
ICD-10	international statistical classification of diseases 10th edition
SNR	signal-to-noise ratio
SinB	speech-in-babble test
PTA	pure-tone audiometry
dB	decibel
RE	right ear
LE	left ear
w	word-based scoring
s	syllable-based scoring
